# Do Leadership, Organizational Communication, and Work Environment Impact Employees’ Psychosocial Hazards in the Oil and Gas Industry?

**DOI:** 10.3390/ijerph19084432

**Published:** 2022-04-07

**Authors:** Gehad Mohammed Ahmed Naji, Ahmad Shahrul Nizam Isha, Abdulsamad Alazzani, Paula Brough, Muhammad Shoaib Saleem, Mysara Eissa Mohyaldinn, Mohammed Alzoraiki

**Affiliations:** 1 Department of Management & Humanities, Universiti Teknologi PETRONAS, Seri Iskandar 32610, Malaysia; shahrul.nizam@utp.edu.my (A.S.N.I.); sh.saleem87@gmail.com (M.S.S.); 2 Department of Accounting and Information Systems, College of Business and Economics, Qatar University, Doha 2713, Qatar; alazzani@qu.edu.qa; 3 School of Applied Psychology, Mount Gravatt Campus, Griffith University, Brisbane, QLD 4122, Australia; p.brough@griffith.edu.au; 4 Department of Petroleum Engineering, Universiti Teknologi PETRONAS, Seri Iskandar 32610, Malaysia; mysara.eissa@utp.edu.my; 5 Department of HRM, College of Administrative and Financial Science, Gulf University, Sanad 743, Bahrain; alzoraiki88@gmail.com

**Keywords:** psychosocial hazards, leadership, organizational communication, work environment, upstream, Malaysian oil and gas

## Abstract

Workplace hazards can have a significant influence on a worker’s physical and mental health, reducing an organization’s effectiveness in terms of safety. However, psychosocial hazards are being recognized as a crucial component that must be addressed for the individual’s and organization’s safety. The purpose of this research was to propose and statistically evaluate a brief theoretical framework based on leadership, organizational communication, work environment, and psychosocial hazards in Malaysia’s upstream oil and gas sector. The framework was tested on 380 Malaysian upstream oil and gas workers. The collected data were analyzed using partial least squares and structural equation modelling (PLS-SEM). The study’s findings revealed that in the Malaysian oil and gas industry, leadership, communication, and work environment negatively influenced the psychosocial hazards. This negative association between predictors and psychosocial hazards, particularly job expectations, control, role, and relationships, indicates new grounds for research. It is discussed how the findings could be used to track employees’ well-being over time and generate focused treatments.

## 1. Introduction

This study tries to answer this main question: do leadership, organizational communication, and work environment have an impact on employees’ psychosocial hazards in the oil and gas industry? To answer this question, we first propose a theoretical framework for enabling an understanding of the effect of these three factors on employees’ psychosocial hazards. Second, we examine the proposed framework for Malaysia’s upstream oil and gas sector. It is essential to determine the effect of leadership, organizational communication, and work environment on psychosocial hazards. We theorize and examine that leadership and organizational communication will reduce psychosocial hazards, which in turn will lead to avoiding any accidents or hazards for oil and gas upstream employees.

The World Health Organization and International Labor Organization [1,2,3] have emphasized that workplace organization, working conditions, workplace interpersonal relationships, and management and design are the key environmental, organizational, and social sources of work-related stress. Psychosocial hazards, in this context, represent a major risk factor that could be hazardous to one’s physical and mental health. Meanwhile, the impacts of psychological risks can be seen at the organizational level [4]. Therefore, work-related stress is a reaction that people experience when they are confronted with work expectations and pressures that are not linked to their skills and talents, challenging their capacity to cope with the stress [5,6]. Furthermore, as a result of exposure to a poor psychosocial workplace and the subsequent work-related stress experience, the issue of exhaustion has become more prevalent. Burnout is characterized in the literature as a state of physical, emotional, and mental tiredness caused by long-term involvement in emotionally taxing work settings [7]. Moreover, workplace environments are seen as stressful when they involve significant work demands that are not well suited to workers’ knowledge, abilities (competencies), or requirements, particularly when employees have little control over their work and receive little support at work [8].

There are several reasons for studying the influence of leadership, organizational communication, and work environment on employees’ psychosocial hazards in the oil and gas industry. Strategic leadership may be a valuable resource in the workplace, as it can mitigate the impacts of psychosocial hazards on employee mental health [9]. Previous research has shown that good leader behaviors, e.g., support, trust, and feedback, are linked to higher worker well-being and can help the employees manage stress [10,11]. Further, people with mental health issues may find it easier to find work if their working environment is improved [12,13]. Failure to do so incurs significant expenses for governments, which must offer social welfare support to persons who would like to work. There is also a developing knowledge that (long-term) joblessness is bad for physical and mental health, so it is reasonable to infer that the contrary is true: work is good for you [14,15]. A study by Westerlund et al. [16] found that when people retire, their fatigue and depression symptoms improve, especially for those who were subjected to some of the worst workplace environments.

Organizational communication refers to personnel and patterns, decision-making structures, structural properties, and the formalization of work processes, while compliance, formal reporting methods, and degrees of cooperation within the business are also factors to consider. Organizational communication has been shown to influence safety behavior and work-related safety outcomes. Effective communication about health and safety issues between managers and employees has been identified as a critical component in the efficacy of safety measures. There is a necessity to study the impact of this issue. The work environment is also seen as an essential factor in determining one’s quality of life. Unemployment, on the other hand, is linked to an increased risk of common mental diseases. Despite the positive effects of labor, an unfavorable psychosocial working environment can be hazardous to workers’ mental wellbeing. A higher pace of work, more highly skilled jobs, and increased use of communication and information technology have all been putting increasing demands on workers’ mental functions.

We focus on Malaysia for several reasons. According to the Malaysia Upstream Summary (2017), the upstream sector of the oil and gas industry is responsible for oil and gas exploration as well as the production of crude oil and natural gas deposits [17]. In Malaysia, according to [18], engineers that work in the oil and gas business learn on the job. It would require ten to fifteen years to acquire all of the skills required for the full spectrum of upstream oil and gas operations. Workers must rotate between several departments or sections within the company. Due to the high exposure to risk in the petroleum business, several risk-reduction strategies and models are required, such as risk-sharing mechanisms in strategic partnerships for the development of hydrocarbon reservoirs [19,20]. Given the complex and hazardous environment of upstream operations, minimizing and preventing hazard exposures in upstream oil and gas places of work is an important quality for safety outcomes [21,22].

To demonstrate current safety performance in upstream oil and gas operations, there are insufficient empirical investigations [6,23]. This is because upstream operations are intertwined and intermingled, resulting in a variety of hazards that are hard to anticipate and regulate. Several dangers arise during exploration and production, e.g., as during drilling, processing, and transportation, environmental risks, gas emissions, and chemicals, all of which pose significant threats to employees’ lives and influence the environment, contributing to the greenhouse effect [24]. Malaysia’s oil and gas sector contributes considerably to the country’s economy, and Malaysia is one of the world’s top LNG exporters [25]. Because of inappropriate handling and safety procedures and safeguards, petrochemical drilling operations are highly associated with health and safety hazards [26]. Psychological risk has become one of the factors contributing to Malaysia’s high accident statistics in the oil and gas industry [27]. Furthermore, the function and consequences of psychosocial hazards at work have been extensively explored [28], considering the impact of psychosocial risk on health management practices on performance. Employers and employees are more likely to have good attitudes toward safety if the number of psychological hazards is reduced [29].

The oil and gas sector is inevitably focused on maintaining a controlled environment [30]. To combat this precarious situation, leadership, organizational communication, and work environments must place a greater emphasis on innovation, cost reduction, and reducing the impact on the environment [31,32]. The oil and gas upstream sector is complex and information-driven, with data quantities growing dramatically to save costs and time [33].

Therefore, this research aims to evaluate a theoretical framework based on leadership, organizational communication, and work environment between employees’ psychosocial hazards and to find the theoretical relationship impacts between the dependent variables and independent variables in Malaysia’s upstream oil and gas sector, as well as to utilize huge information to enhance operational knowledge among employees in the upstream oil and gas industry and to assist basic work environment, organization communication, and leadership in various upstream sector procedures. This study finds that there is an impact of leadership, organizational communication, and work environment on psychosocial hazards.

## 2. Literature Review

### 2.1. Leadership

Leaders must understand how to enhance safety regulations in their firms as accidents occur for a variety of causes. Several studies have shown that certain leadership practices improve safety [34,35]. In healthcare companies, leadership has been acknowledged as a significant factor in enhancing quality and performance [36]. The impact of leadership on an organization’s ability to innovate is a hot topic right now. Leadership helps innovation in general, and the role of leadership in the growth of innovation in the oil and gas sector has been highlighted in particular [37]. Leadership can actively influence an organization’s inventive potential or the creation of conditions that encourage innovation, particularly those connected to organizational learning.

Strategic leadership may be a valuable resource in the workplace, as it can mitigate the impacts of psychosocial hazards on employee mental health [9]. Previous research has shown that good leader behaviors, e.g., support, trust, and feedback, are linked to higher worker well-being and can help employees to manage stress [10,11]. Over time, the organization’s attitudes and beliefs will transition from removing physical risks to removing work demands prone to human error by implementing systems that proactively enhance workplace conditions, to the point where safety becomes a core value and an inherent component of operations [11,38]. The safety leader is an additional component required for this to happen. Managers at all levels must lead by example when it comes to workplace safety. Some people are born with some of the characteristics that we identify with leadership. This does not, however, rule out the possibility of other people serving as safety leaders. It is behavior, more than personality, that distinguishes a person as a leader [39].

Leadership that values both production and people has been shown to achieve the best results [40]. It has been proven that leadership that values both productivity and people has the best results in motivating people to perform. In addition, to create a strong safety culture, executives must employ the following leadership styles:

Transactional vs. transformational leadership: To put it simply, transformational leaders are managers who set goals, track progress, and make adjustments as needed. Transformation leaders have a vision and motivate others to pursue it well beyond their personality [41,42]. While safety leaders require transactional skills, they are unable to drive employee satisfaction without transformational talents.

Situational and contextual [43]: Good leaders adjust their leadership style according to the situation at hand and the context in which the organization operates (branch of industry, country, and so on). This is true for a safety leader as well.

Psychological harm in the workplace as a result of psychosocial risk is a severe work health and safety issue [44]. Work-related psychological harm comes at a high price. There are considerable costs for organizations in terms of disease and absence performance, in addition to the personal pain of psychological injury. For instance, Dollard, et al. [44] posit that safety-specific leadership has a considerable impact on psychosocial risk.

**Hypothesis** **1** **(H1).** *Leadership for safety will have a declining effect on psychological hazards.*

### 2.2. Organizational Communication

Organizational communication is described as the method through which the organization communicates details about the environment and the employee’s task to its workers [45]. The goal of organizational communication is to convey information to employees in a timely, essential, and relevant manner so that everyone is informed about their job and the environment [46]. Simply put, communication refers to the mechanism for exchanging information as well as the environment in which it occurs inside an organization. Moreover, employee safety requires not only a focus on worker safety culture, but also initiatives to improve organizational characteristics and culture through organizational change [47]. Furthermore, organizational communication personnel and patterns, decision-making structure, structural properties, formalization of work process processes and compliance, formal reporting methods, and the degree of cooperation within the business are also factors to consider. These have been shown to influence safety behavior and work-related safety outcomes [48,49].

In addition, employees’ perceived risk, risk attitude, safety improvement ideas, safety training courses, policy communications, and safety committees are all examples of passive measurement. Self-reported rates of accident and occupational injuries can be used to describe safety performance [50]. Huang et al. [51] investigated safety in a variety of workplaces, including manufacturing, construction, service, and transportation, measuring safety performance, worker safety control, and self-reported effect on organization culture or the atmosphere of the organization [52,53].

Communication rules have also been investigated concerning psychological risks by Johari et al. [54]. Effective communication about health and safety issues between managers and employees has been identified as a critical component in the efficacy of safety measures. There is continuous research that suggests a strong link between communication quality and occupational accidents [55,56].

Health and safety objectives must be incorporated into organizations with the necessary health and safety targets at the senior management level and included in all team meetings agendas [57]. Any evaluation and intervention must be communicated throughout the organization to reflect management’s commitment to resolving the problem [58]. This is also critical to the logistics of risk management process execution and, in particular, to ensure proper worker engagement. According to the findings of Way [59], there is a favorable relationship between communication and psychological risks.

**Hypothesis** **2** **(H2).** *Organizational communication has a negative association with psychosocial hazards.*

### 2.3. Work Environment

According to Pawirosumarto [60], a work environment is a place where employees accomplish their tasks, and it can have both positive and negative consequences on the employees’ ability to fulfil their goals. A positive work environment will have a positive effect on employment continuity, whilst a poor work environment will hurt job continuity. According to Awan [61,62], a working environment is one in which employees collaborate to achieve organizational goals.

A person’s work environment can be defined as the place where they work, as well as the surrounding milieus. It is the social and professional setting in which an individual is expected to engage with a variety of people. Employees’ overall productivity is influenced by their working environment. We define work environment as those procedures, systems, structures, tools, or environments in the workplace that influence individual performance favorably or negatively [63]. Rules, policies, culture, working relationships, resources, work locations, and internal and external environmental elements all influence how participants perform their work roles in the workplace [64,65]. Employee performance and productivity are heavily influenced by their working environment. Processes, procedures, structures, tools, or situations in the workplace that influence individual performance favorably or negatively are referred to as the work environment [66,67].

Work is seen as an essential factor in determining one’s quality of life. Unemployment, on the other hand, is linked to an increased risk of common mental diseases [68]. Despite the positive effects of labor, an unfavorable psychosocial working environment can be hazardous to workers’ mental wellbeing. A higher pace of work, more high-skilled jobs, and increased use of communication and information technology have all been putting increasing demands on workers’ mental functions [69,70]. According to van den Heuvel et al. [71], there is a link between the work environment and psychological dangers.

**Hypothesis** **3** **(H3).** *The work environment has a negative association with psychosocial hazards.*

### 2.4. Psychosocial Hazard

Workplace psychosocial hazards are typically linked to work-related stress [3,72,73], which has been linked to individual health problems, such as depression, musculoskeletal illnesses, and heart disease in longitudinal studies [74,75,76]. Many types of research have linked psychosocial hazards to organizational outcomes, such as presenteeism, lost time, absenteeism, work–family conflict, job satisfaction, and intention to leave the organization [5,77,78,79]. According to [21], workplaces where employees are faced with high demands and little control, as well as insufficient support by management and/or coworkers, are considered to be highly stressful [80]. Despite increased knowledge of the impact of psychosocial risks and related concerns in the oil and gas business, company-level initiatives to address psychosocial risks are minimal, if not non-existent in many situations [81]. Since the beginning of the oil and gas business, health, safety, and the environment (HSE) have been an integral aspect of operational risk management. As a result, a varied team of industry professionals undertook an additional study, including representatives from operating firms, well drilling and maintenance companies, and industry trade associations [82].

Psychosocial hazards have the greatest influence on the mental health of health care employees of all the different categories of hazards [83]. Psychosocial risks are those characteristics of job design, structure, and management that have the potential to cause emotional or physical injury, as well as their social context [78]. Interpersonal interactions at work, work overload, work stress, limited job control, bullying, violence, and poor organizational justice are all examples of work-related psychosocial hazards. Long-term exposure to these psychosocial risks has been linked to an increased risk of health problems, such as heart disease, and may also lead to psychiatric illnesses such as anxiety [84].

Furthermore, in Malaysia’s industrial sector, the identification of psychosocial hazards and work-related stress is still in its infancy. By the year 2015, Malaysia’s Department of Occupational Safety and Health and the Social Security Organization had only documented four industrial instances as “psychosocial issues” under “types of illnesses” and 344 as mental health cases. The majority of other occupational hazards, such as physical, chemical, and biological agents, as well as a variety of other environmental factors, are still a primary focus in Malaysian companies [85].

**Figure 1:**
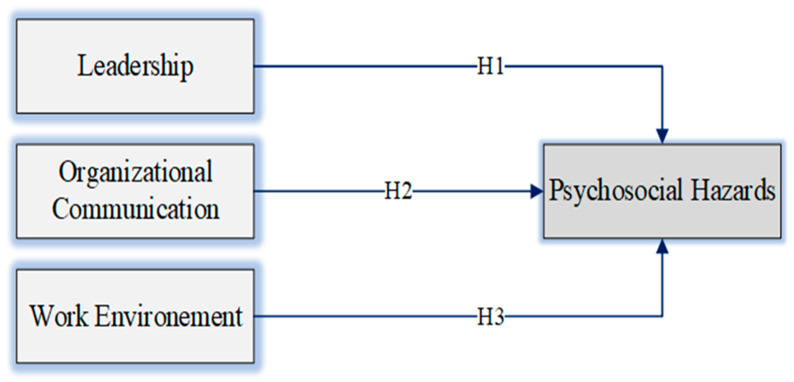
Conceptual Model.

As shown in Figure 1, a proposed framework in this study mentioned that good leadership, organizational communication, and work environment will reduce the psychosocial hazards among employees in the oil and gas sector.

Therefore, leadership, organizational communication, and work environment are the independent variables. This research will determine the relationship between the independent variables and psychosocial hazards. The current study will also be focusing on reducing the mental stress which causes harm to the employees. The outcomes will contribute to a better understanding of the factors that can influence the employee’s safety performance and how to enhance a positive safety attitude towards workers.

## 3. Methodology

### 3.1. Instruments Design

The instruments for this research are organized into two sections: (a) demographic data and (b) elements for calculating the autonomous, moderating, and dependent variables. The items [86,87,88] were made using previous studies. The statements were rated on a five-point Likert scale that ranged from “Never” (1) to “Always” (5). As a result, Table 1 shows the demographic information of the respondents.

### 3.2. Pre-Testing Method

We used two basic pre-testing strategies to evaluate our questionnaire survey before data collection. The survey design was validated in two phases: content validity and construct validity, where the recommended guideline for pre-test is as follows:

Reliability: Two reliability criteria were applied to this survey questionnaire.

Cronbach’s Alpha, where Alpha ≥ 0.70 (acceptable) Alpha ≥ 0.80 (Good) Alpha ≥ 0.90 (Excellent) [89].

Composite reliability: CR ≥ 0.80 (Acceptable) [90].

Content validity: Content validity is determined by how well the indicators convey the construct’s category content. The in-depth analysis indicates how closely a single component resembles the concept under consideration [90].

Construct validity:

Confirmatory factor analysis (CFA)

Factor loading ≥ 0.60

### 3.3. Questionnaire Design

A comprehensive, cross-sectional survey has been developed for this study. According to the pilot study (preliminary test), appropriate modifications have been made. To investigate the influence of leadership, organizational communication, and work environment on psychosocial hazards, the main survey was distributed to a large number of potential upstream oil and gas employees. We have utilized the Morgan Table methodology analysis to determine the sample size [91]. According to Yin [92], the SEM sample size must be more than 100, although Kline [93] feels that a comprehensive path model requires at least, 200 samples. We have received 380 responses from the workers in the upstream oil and gas sector in Malaysia. This questionnaire is divided into two sections: part one contains demographic information about the respondents, and part two contains elements meant to evaluate two variables using a five-point Likert scale ranging as “(1 = Never, 2 = Seldom, 3 = Neutral, 4 = Often, 5 = Always)” [94,95] as well as instruments modified from prior studies [86,87,88].

This study analyzes the relationship between leadership, organizational communication, work environment, and psychosocial hazard in the oil and gas sector in Malaysia. By evaluating the framework constructs in the Malaysian context, this research examined alternative ways for reducing risk and incident mitigation. Appendix A shows the questionnaire structure.

### 3.4. Partial Least Squares-Structural Equation Modelling (PLS-SEM)

The variance-based SEM partial least squares-structural equation modelling (PLS-SEM) is used to evaluate composite-based path models. PLS-SEM is first used to assess the theoretical model and hypothesized correlations. PLS-SEM good for delving into a theory and studying a complicated model with multiple latent variables [96,97]. It can also accommodate single-item structures and small samples [98]. Smart-PLS software is extensively used to execute PLS-SEM, and it may be successfully implemented in a variety of business and marketing contexts, such as tourism research. Even though there are certain flaws in the PLS approach in applied research, several scholars believed that it should be used in social science disciplines [99,100,101,102,103,104]. Meanwhile, it has been stated that ignoring the positive aspects of PLS is not a good approach, because the majority of its flaws are not attributable to issues with the technique itself. Given the benefits of PLS-SEM in terms of consistency, reliability, and validity in travel and tourism sector research, it is the best strategy for this study to use to assess the hypotheses and conceptual basis proposed. PLS-SEM, on the other hand, may oversimplify a complex decision-making system. Many scholars have advised the use of computational intelligence techniques to overcome this limitation [105,106,107]. It is worth noting that the PLS-SEM method uses boot-strapping to normalize the indications at first [108,109].

Finally, the three-hypothesis provided in this study were evaluated by using the PLS-SEM approach. Variance inflation factor (VIF) was utilized to investigate multicollinearity challenges to evaluate multicollinearity [110]. This was performed by evaluating the assessment model’s fittings and path analyses utilizing the Smart-PLS V3.2.1 software (Ringle, C.M.; Boenningstedt, Germany, 2015) [111,112]. Harmans’ single factor has been examined using the SPSS (Ringle, C.M.; Boenningstedt, Germany, 2015) tool, to measure common method bias [113].

## 4. Result

### 4.1. Measurement Model

Since the data gathered by questionnaire surveys are generally normal, the research study used a partial least square, Smart-PLS version3.0, as a statistical technique to explore the structural and measurement model, as conducted in a previous study [98]. We evaluated the risks of common technique bias by examining the data’s complete collinearity, as proposed by previous studies [86,88] because the data came from a specific source. In the entire collinearity test, all variables have been regressed against a common variable, indicating that if VIF values are below those shown in Table 2 there is no bias from the specific source data collection. Therefore, based on our findings, all VIF values were below those in Section 3.3. As a result, our research shows that single-source businesses are not a big issue. The results of the comprehensive collinearity test are provided in Table 2.

#### Framework Measurement

**Figure 2:**
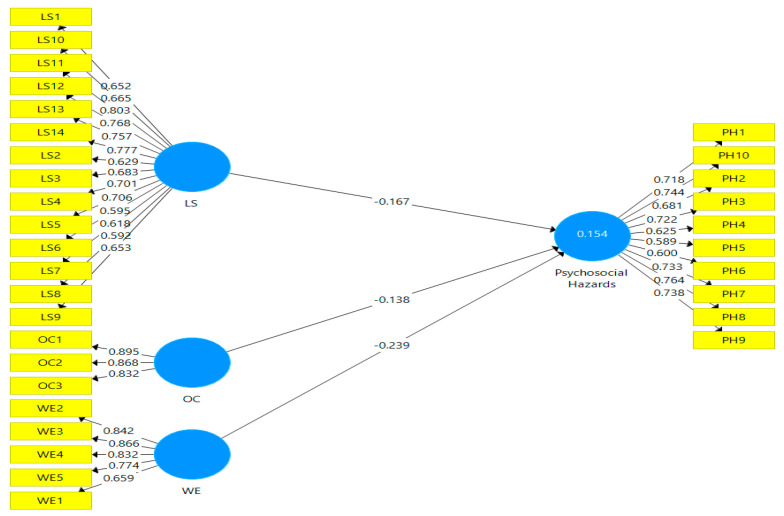
Structure model outcomes and $R^{2}$ values.

**Figure 3:**
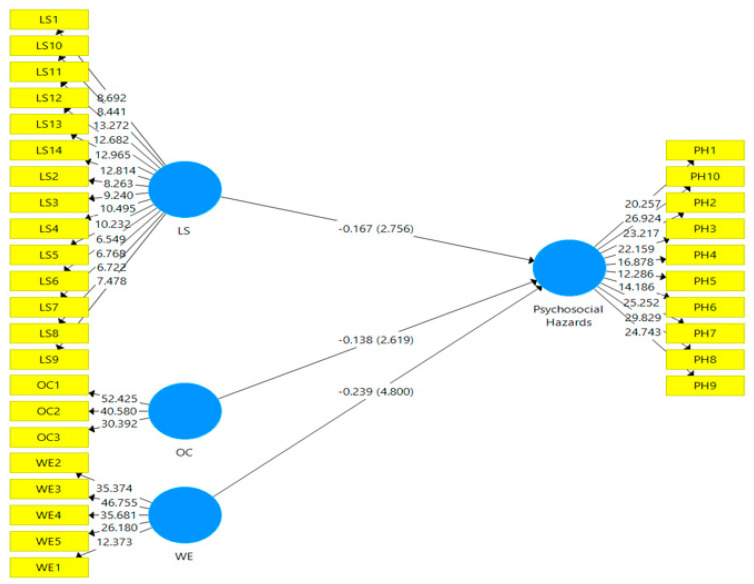
Bootstrapping analysis and T-values.

Smart-PLS utilized the partial least squares technique to measure the framework. For the evaluation of the measurement model, two types of validities (convergent and discriminant) were used [96]. SEM is used to illustrate the conceptual framework of the current study for both PLS-Algorithm and bootstrapping analysis, as shown in Figure 2 and Figure 3.

### 4.2. Convergent Validity

The outer loadings (OL), Cronbach’s alpha (CA), construct reliability (CR), average variance extracted (AVE), and validity of the measurement model were assessed initially. The values obtained for all constructs of VIF are listed in Table 1. The values of all indicators should be greater than or equal to the relevant threshold value. Composite reliability (CR) is used to assess internal consistency, and it should be greater than the minimum threshold value of 0.70 [114]. Composite reliabilities were used to determine internal consistency. The suitability of a construct is demonstrated by the value of the average variance extracted (AVE) being greater than 0.05 [115]. For newly developed items, the factor loading for every item should exceed 0.5. For established items, the factor loading for every item should be 0.6 or higher [116]. Any item having a factor loading less than 0.6 and an R2 should be greater than 0.1 based on the work of Falk and Miller [117]. Therefore, the correlation coefficients for all constructs are more than 0.50, showing that they are dependable for the contextual measures without affecting the hypothesis. Table 3 further demonstrates that the AVE values for all constructs are greater than 0.5, demonstrating sufficient convergent validity.

### 4.3. Discriminant Validity

Discriminant validity reflects the statistical and theoretical disparities between each pair of constructs [115,118]. To assess the discriminating construction’s validity, the AVE square roots of each construct can be compared to the associations between them. According to Fornell and Larcker [119], AVE should be greater than the correlation between latent variables. The discriminating validity of the measurement model is demonstrated by the outcomes in Table 4.

### 4.4. Discriminant Validity (Cross-Loading)

In cross-loadings, the authors looked at several things to see which ones have higher contents on the same construct and which ones have high loadings on many constructions. This approach aims to assess if the loading of indicators on a specific latent variable should be greater than the loading of indicators on other latent variables per line. This implies that the loading of indications or objects on the primary construct must be higher than on other constructs. Table 3 shows that all latent indicators (variables) have a larger loading than in another construct, row by row. The results show a significant level of one-dimensionality for each construct.

### 4.5. Structural Model (Path Analysis)

Path analysis is a statistical technique for describing the directed connections between a set of variables. Path model is analogous to multiple regression, correlation analysis, factor analysis, discriminant analysis, and more comprehensive families of models in multivariate analysis of variance and covariance analyses (MANOVA, ANOVA, ANCOVA) [120]. Following the fitting of the model, structural equation modelling can be used to investigate the relationship among variables. The structural model describes the relationships between research variables in considerable detail [121].

The findings show the relationship between exogenous and endogenous (independent and dependent) variables. The fit of the whole model, with hypothesized estimates coefficients, dimensions, route, and relevance, is the primary emphasis of structural model evaluation [122]. PLS-SEM was used to explore the impact of leadership-organizational communication-work environment on psychosocial hazards in this model, which was performed following the research context. The accompanying hypothesis model is shown in Figure 2 and Figure 3 shows how the bootstrapping approach was used to determine the significance hypothesis model through T-value. The process of bootstrapping is used to generate samples that seem to be equal to the actual data during the random reconsideration of the original information. This technique not only assesses the data’s dependability, but also anticipates the significance and inaccuracy of the obtained path coefficient [122]. Endogenous constructs were examined for standardized path coefficients β and t-values, path significance, and $R^{2}$, as shown in Figure 2 and Figure 3. Endogenous constructs were examined for standardized path coefficientsβ and t-values, path significance, and $R^{2}$, as shown in Figure 2 and Figure 3.

As a result of the bootstrapping method, Table 5 and Table 6 show the *p*-values for the study path. Leadership had a negative association with psychosocial hazards according to the data (β = −0.167, t 2.88), while organizational communication had a negative association with psychosocial hazards based on the data (β = −0.138, t 2.69), and work environment had a negative influence on psychosocial hazards, as shown in Table 6 below (β = −0.239, t 4.75). Therefore, all three hypotheses had a favourable and significant influence between exogenous and endogenous variables.

#### Construct Cross Validated Communality Q² & Bootstrapping R Square

For the model’s endogenous variables, the PLS technique provided multiple squared (R^2^) correlations. R^2^ is regarded as standard regression in the SEM-PLS technique. The total variance is defined as the R^2^ [111]. The explanation of exogenous variables in the endogenous variables has proved the R^2^. As a result, a higher R^2^ value improves the structural model’s prediction power. The R^2^ values in this investigation were determined using the PLS technique, as shown in Table 7. The dependent variable (psychosocial hazards) had an R^2^ value of 0.154 in this model, indicating that the latent independent variable (Leadership-organizational communication-work environment) could explain 15.4% of psychosocial hazards. According to Chin [123], the R^2^ result of 15.4% indicates that (leadership–organizational communication–work environment) has a significant impact on psychosocial hazards.

A structural model’s capacity to determine the model’s predictive significance is an important characteristic. Cross-validated redundancy analyses for the endogenous variables were monitored using the blindfolding approach. The results revealed that the Q^2^ (0.363) project performed greater than 0, indicating that the independent construct in this study was statistically significant for the dependent construct [124]. The findings of Q^2^ are also shown in Table 7, which is greater than 0, implying that the model has strong predictive relevance (significant).

## 5. Discussion

Through this study, we tested the direct relationship between organizational communication → psychosocial hazards, work environment → psychosocial hazards, and leadership → psychosocial hazards. Our finding supported our assumption that there was a significant relationship between our three proposed predictors and psychosocial hazards at the workplace. Improving leadership, organizational communication, and work environment among upstream oil and gas employees can substantially reduce accidents for the workplace employees, as most accidents are the outcome of psychological strain and stress. The statistical data and analysis of SEM modelling give a solid foundation for understanding relationships between various variables. During the evaluation and interpretive process, some significant findings emerged.

It is important to determine the effect of leadership, organizational communication, and work environment on psychosocial hazards to reduce and avoid any accidents or hazards of oil and gas upstream employees [125,126]. The statistics have been used to investigate the impact of leadership, organizational communication, and work environment on psychosocial hazards. According to the data, improving the leadership, organizational communication, and work environment among employees in the oil and gas sectors will reduce the incidents and accidents in their workplace. 

Furthermore, when the values of (β = −0.167, −0.138, −0.239) accordingly, leadership, organizational communication, and work environment have a significant negative influence on psychosocial hazards. The findings suggest that a better leadership, organizational communication, and work environment would aid employees in managing their job demands, remaining focused, avoiding weariness, and maintaining a good response time while they are working in their workplace. These measures will keep workers safe from any injuries or accidents in the workplace environment. This finding is consistent with Jiang [127], who agreed that implementing improvements in leadership, organizational communication, and work environment would improve employees’ workplace safety, whereas, at the moment, leadership, organizational communication, and work environment among employees are influenced more by the company’s commitment to managing their safety and training employees to avoid hazardous and accidents. Furthermore, one of the most essential techniques for reducing accidents in construction projects is improving leadership, organizational communication, and work environment [128,129]. As a result, it is unsurprising that in the oil and gas industry, psychosocial hazards are essential for increasing the number of accidents in the workplace [69,130].

We can deduce from the aforementioned findings that the leadership style and its output will affect psychosocial hazards, organizational communication will influence psychosocial hazards, and the work environment will also have an impact on psychosocial hazards. Employees’ commitment to the health and safety of employees during their duties is determined by the level of occupational and health safety, which is defined in terms of the leadership organization, organizational communication, and work environment of worker behaviour. In the current study, all leadership, organizational communication, and work environment data met expectations. The study’s goal was met, and it was consistent with earlier research [58,131,132,133].

## 6. Conclusions

Psychosocial hazards have a significant and negative impact on organizations due to employee health and behaviour, which are both linked to a variety of organizational outcomes. As a result, companies must have procedures and instruments in place to deal with this sort of risk, and the risk management process must be incorporated into the organization’s management systems. Many sectors place a premium on leadership, organizational communication, and work environment and the oil and gas industry, like many others, has seen employee accidents as a result of a poor safety environment. The PLS-SEM approach has been used to determine the effect of leadership, organizational communication, and work environment on psychosocial hazards. A direct path has been evaluated in the created model using data from the Malaysian upstream oil and gas sector. Furthermore, by examining the link between variables, the direct path among variables has been established. The findings concur that instilling leadership, organizational communication, and work environment in employees can improve their safe environment and help to reduce the likelihood of work environment accidents and fatalities.

This research has many contributions to offer. First, the study adds to the body of knowledge in the oil and gas upstream sector by enhancing the knowledge of leadership, organizational communication, and work environment. Second, the research results provide a framework for future study by demonstrating that interest in leadership, organizational communication, and work environment has a significant and negative effect on psychological hazards. The recent study also provides a clear picture for managers of upstream oil and gas firms who want to help their employees succeed in their jobs by focusing on their safe environments. By focusing on employers’ attention and alertness during oil and gas operations, the current study can benefit all relevant parties, supervisors, and employees through engagement in leadership, organizational communication, and the work environment.

### Study Limitations

Despite the study’s contributions, it is noted that there are several intrinsic constraints relating to data collection and generalization. First, as compared to theory-building methodologies, this will not reflect a comprehensive understanding of the situation, as cross-sectional research or single image survey research do, as in the case of Malaysian upstream oil and gas industry employees. As a result, it is recommended that higher sample sizes be used. Second, the survey participants were all oil and upstream workers from Malaysia. Because the findings may not be generalizable to other developing countries, more research should be conducted in other parts of southeast Asia. Third, while our study focused on oil and gas upstream employees, given the expanding volume of global trade and production, the relevance of exploration and production should not be overlooked [134,135]. Other types of production and exploration, including drilling, optimization, and well logging interpretation, should be included in future research, as well as the issues they confront. Similarly, more research on worker concentration and other related variables that influence employee performance is needed. Finally, the study’s methodology is mitigated by the fact that it has been conducted using self-reported surveys. An experimental strategy to measure employee vigilance and reaction time would be preferable for future research.

## Figures and Tables

**Table 1:** Demographic Information.

Demographic Categories	Categories	Frequencies (*n* = 380)	Percentages %
Gender	Male	376	98.95%
	Female	4	1.05%
Age	20–29 Years	49	12.89%
	30–39 Years	164	43.16%
	40–49 Years	94	24.74%
	50–59 Years	58	15.26%
	60 years and above	15	3.95%
Marital Status	Single	59	15.52%
	Married	293	77.11%
	Divorced	28	7.37%
Education	Graduate/Postgraduate	16	4.21%
	College	55	14.47%
	Secondary	29	77.89%
	Primary	13	3.43%

**Table 2:** Collinearity Statistics (VIF) Results.

LS	OC	WE
**1.175**	1.174	1.086

**Table 3:** The outcomes of convergent validity and reliability.

Constructs	Items	OL	CA	CR	AVE
**Leadership**	LS1	0.652	0.92	0.926	0.574
	LS2	0.629			
	LS3	0.638			
	LS4	0.701			
	LS5	0.706			
	LS6	0.595			
	LS7	0.618			
	LS8	0.592			
	LS9	0.653			
	LS10	0.665			
	LS11	0.803			
	LS12	0.768			
	LS13	0.757			
	LS14	0.777			
**Organizational Communication**	OC1	0.895	0.832	0.899	0.749
	OC2	0.868			
	OC3	0.832			
**Work** **Environment**	WE1	0.659	0.854	0.897	0.637
	WE2	0.842			
	WE3	0.866			
	WE4	0.832			
	WE5	0.774			
**Psychosocial** **Hazards**	PH1	0.718	0.88	0.902	0.582
	PH2	0.681			
	PH3	0.722			
	PH4	0.625			
	PH5	0.589			
	PH6	0.6			
	PH7	0.733			
	PH8	0.764			
	PH9	0.738			
	PH10	0.744			

**Table 4:** Discriminant Validity.

Constructs	Leadership	Organizational Communication	Psychosocial Hazards	Work Environment
**Leadership**	0.689			
**Organizational** **Communication**	0.353	0.865		
**Psychosocial Hazards**	−0.271	−0.252	0.694	
**Work Environment**	0.232	0.231	−0.309	0.798

**Table 5:** Cross-loading outcomes of discriminate validity.

Items	Leadership	Organizational Communication	Psychosocial Hazards	Work Environment
**LS1**	0.652	0.183	−0.114	0.126
**LS10**	0.665	0.247	−0.129	0.174
**LS11**	0.803	0.336	−0.307	0.195
**LS12**	0.768	0.286	−0.243	0.158
**LS13**	0.757	0.327	−0.235	0.205
**LS14**	0.777	0.31	−0.26	0.162
**LS2**	0.629	0.161	−0.143	0.113
**LS3**	0.683	0.135	−0.134	0.143
**LS4**	0.701	0.229	−0.177	0.127
**LS5**	0.706	0.197	−0.169	0.137
**LS6**	0.595	0.213	−0.084	0.223
**LS7**	0.618	0.193	−0.126	0.173
**LS8**	0.592	0.217	−0.119	0.166
**LS9**	0.653	0.204	−0.089	0.195
**OC1**	0.33	0.895	−0.241	0.223
**OC2**	0.261	0.868	−0.196	0.203
**OC3**	0.32	0.832	−0.214	0.17
**PH1**	−0.218	−0.188	0.718	−0.2
**PH10**	−0.176	−0.173	0.744	−0.252
**PH2**	−0.212	−0.144	0.681	−0.182
**PH3**	−0.198	−0.202	0.722	−0.279
**PH4**	−0.196	−0.125	0.625	−0.184
**PH5**	−0.013	−0.12	0.589	−0.178
**PH6**	−0.165	−0.133	0.600	−0.123
**PH7**	−0.232	−0.194	0.733	−0.199
**PH8**	−0.226	−0.227	0.764	−0.251
**PH9**	−0.181	−0.201	0.738	−0.253
**WE2**	0.202	0.152	−0.253	0.842
**WE3**	0.125	0.171	−0.24	0.866
**WE4**	0.253	0.223	−0.23	0.832
**WE5**	0.202	0.175	−0.273	0.774
**WE1**	0.139	0.204	−0.229	0.659

**Table 6:** Hypothesized testing outcomes.

Hypotheses	Path	Beta-Value (*n* = 380)	ST	T-Value	*p*-Values
**H1**	Leadership -> Psychosocial Hazards	−0.167	0.058	2.88	Significant
**H2**	Organizational Communication -> Psychosocial Hazards	−0.138	0.051	2.69	Significant
**H3**	Work Environment -> Psychosocial Hazards	−0.239	0.05	4.75	Significant

**Table 7:** R2 and Construct Cross validated Communality Q².

	R Square	R Square Adjusted	SSO	SSE	Q² (= 1 − SSE/SSO)
Leadership	-	-	5320	3172.485	0.404
Organizational Communication	-	-	1140	599.138	0.474
Psychosocial Hazards	0.154	0.147	3800	2419.288	0.363
Work Environment	-	-	1900	1040.263	0.452

## Data Availability

We are continually working on this project, and the data will be used for future research and analysis. However, any researcher who needs the data for further investigations can contact the corresponding author through email with reasonable justification.

## Appendix A. Questionnaire Structured

**Table A1:** Research instrument.

Contracts	Codes	Items	References
Leadership	LS1	“My senior managers/leaders have established a safety responsibility system.”	[86]
	LS2	“My senior managers/leaders express an interest in acting on safety policies.”	
	LS3	“My senior managers/leaders are concerned about safety improvement.”	
	LS4	“My senior managers/leaders establish clear safety goals.”	
	LS5	“My senior managers/leaders coordinate with other departments to solve safety issues.”	
	LS6	“My senior managers/leaders explain the safety mission clearly.”	
	LS7	“My senior managers/leaders encourage workers to provide safety suggestions.”	
	LS8	“My senior managers/leaders emphasize worksite safety.”	
	LS9	“My senior managers/leaders stress the importance of wearing personal protective equipment.”	
	LS10	“My senior managers/leaders encourage workers’ participation in safety decision-making.”	
	LS11	“My senior managers/leaders encourage workers to report potential incidents without punishment”	
	LS12	“My senior managers/leaders show consideration for workers.”	
	LS13	“My senior managers/leaders trust workers.”	
	LS14	“My senior managers/leaders praise workers’ safety behavior.”	
Organizational communication	OC1	“There is good communication here about safety issues which affects me.”	[87]
	OC2	“Safety information is always brought to my attention by my line manager/supervisor.”	
	OC3	“My line manager/supervisor does not always inform me of current concerns and issues.”	
Work Environment	WE1	“Operational targets often conflict with safety measures.”	[87]
	WE2	“Sometimes I am not given enough time to get the job done safely.”	
	WE3	“Sometimes conditions here hinder my ability to work safely.”	
	WE4	“There are always enough people beside me to get the job done safely.”	
	WE5	“I cannot always get the equipment I need to do the job safely.”	
Psychosocial Hazards	PH1	“Is your workload unevenly distributed so it piles up?”	[88]
	PH2	“How often do you not have time to complete all your work tasks?”	
	PH3	“Do you get behind with your work?”	
	PH5	“Do you have to work very fast?”	
	PH5	“Is it necessary to keep working at a high pace?”	
	PH6	“Does your work require you to make difficult decisions?”	
	PH7	“How often have you had problems relaxing?”	
	PH8	“How often have you been irritable?”	
	PH9	“How often have you been tense?”	
	PH10	“How often have you been stressed?”	
